# TMEM16A inhibition suppresses melanoma metastasis

**DOI:** 10.3724/abbs.2025133

**Published:** 2025-08-05

**Authors:** Na Zhou, Chuangxin Pei, Xue Lu, Peng Shi, Siqi Wu, Huaqun Chen

**Affiliations:** Jiangsu Key Laboratory for Molecular and Medical Biotechnology College of Life Sciences Nanjing Normal University Nanjing 210023 China

As a highly aggressive malignancy arising from melanocytes, malignant melanoma accounts for the majority of skin cancer-related deaths worldwide. Metastases, particularly lung and brain metastases, contribute significantly to mortality [1]. Although targeted therapy (BRAF/MEK inhibitors) and immunotherapy (checkpoint inhibitors) have greatly improved the overall survival of patients, drug resistance and toxicity remain major clinical challenges [ 2, 3] . Therefore, exploring new approaches to combat melanoma metastasis is imperative. Melanoma metastasis involves multiple processes, including phenotype switching (epithelial-mesenchymal transition, EMT), migration, invasion and infiltration [4]. Phenotype switching occurs at the early stage of metastasis and is characterized by the downregulation of epithelial markers ( *e*. *g*., E-cadherin) and the upregulation of mesenchymal markers ( *e*. *g*., N-cadherin and Vimentin). Metastasis depends on highly regulated and complex remodeling of the tumor microenvironment formed by cells as well as by biochemical and biophysical components of the extracellular matrix (ECM) and their intricate interactions within and around a solid tumor mass [5]. These processes are primarily mediated by the altered expression of metastasis-associated genes, and targeting the expression of these genes may be a promising strategy for inhibiting melanoma metastasis.

TMEM16A (also known as ANO1), a calcium-activated chloride channel (CaCC) localized to the plasma membrane and organelle membranes, is widely expressed in tissues such as airways, smooth muscles, and neurons, where it plays important physiological roles in regulating smooth muscle contraction and chloride ion secretion [6]. Growing evidence indicates that TMEM16A is overexpressed in various cancers and contributes to tumor progression by increasing cell proliferation, invasion, and metastasis [7]. The expression level of TMEM16A is closely related to tumor size and differentiation, is associated with advanced stage and poor prognosis, and can even be used as a biomarker for certain malignant tumors [8]. We previously observed a high expression level of TMEM16A in a human melanoma cell line, A375, which harbors a BRAF V600E mutation, and demonstrated its role in promoting tumor growth [9]. Here, we further showed that elevated TMEM16A expression contributes to melanoma metastasis.

To assess the role of TMEM16A in the migration of melanoma cells, we first performed a wound healing assay. The results revealed that A375 cells with *TMEM16A* knockdown (shT16A) via infection with shRNA lentivirus migrated significantly slower than control cells infected with negative control shRNA (shNC) did at 24 h and 48 h after wound creation (24 h: the healing rate was 38.5% ± 3.3% of shNC, *P*  < 0.001; 48 h: the healing rate was 53.8% ± 2.7% of shNC, *P*  < 0.001) ( Figure 1A,B), indicating that TMEM16A promotes melanoma cell migration. This finding was confirmed by transwell migration assays, where the migration rate (migrated cells/total cells) of shT16A cells was significantly reduced to 61.9% ± 1.6% of that of shNC controls ( *P*  < 0.05; Figure 1C–E). To assess the role of TMEM16A in the invasion of melanoma cells, we conducted a transwell invasion assay. Notably, the relative invasion (invaded cells/total cells) of shT16A cells significantly decreased to 67.5% ± 6.1% of that of shNC controls ( Figure 1D,F; *P*  < 0.01), suggesting that TMEM16A also enhances cell invasion. We next examined the role of TMEM16A in cell adhesion, as cancer cell-ECM adhesion critically contributes to tumor colonization [10]. Compared with shNC cells, shT16A cells exhibited significantly impaired adhesion to Matrigel ( Figure 1G; *P*  < 0.01). As these effects were similarly inhibited by TMEM16A inhibitors (T16inh-A01 and Caccinh-A01) ( Figure 1H–K), our results collectively establish TMEM16A as a key mediator of these metastatic processes.

**Figure 1:**
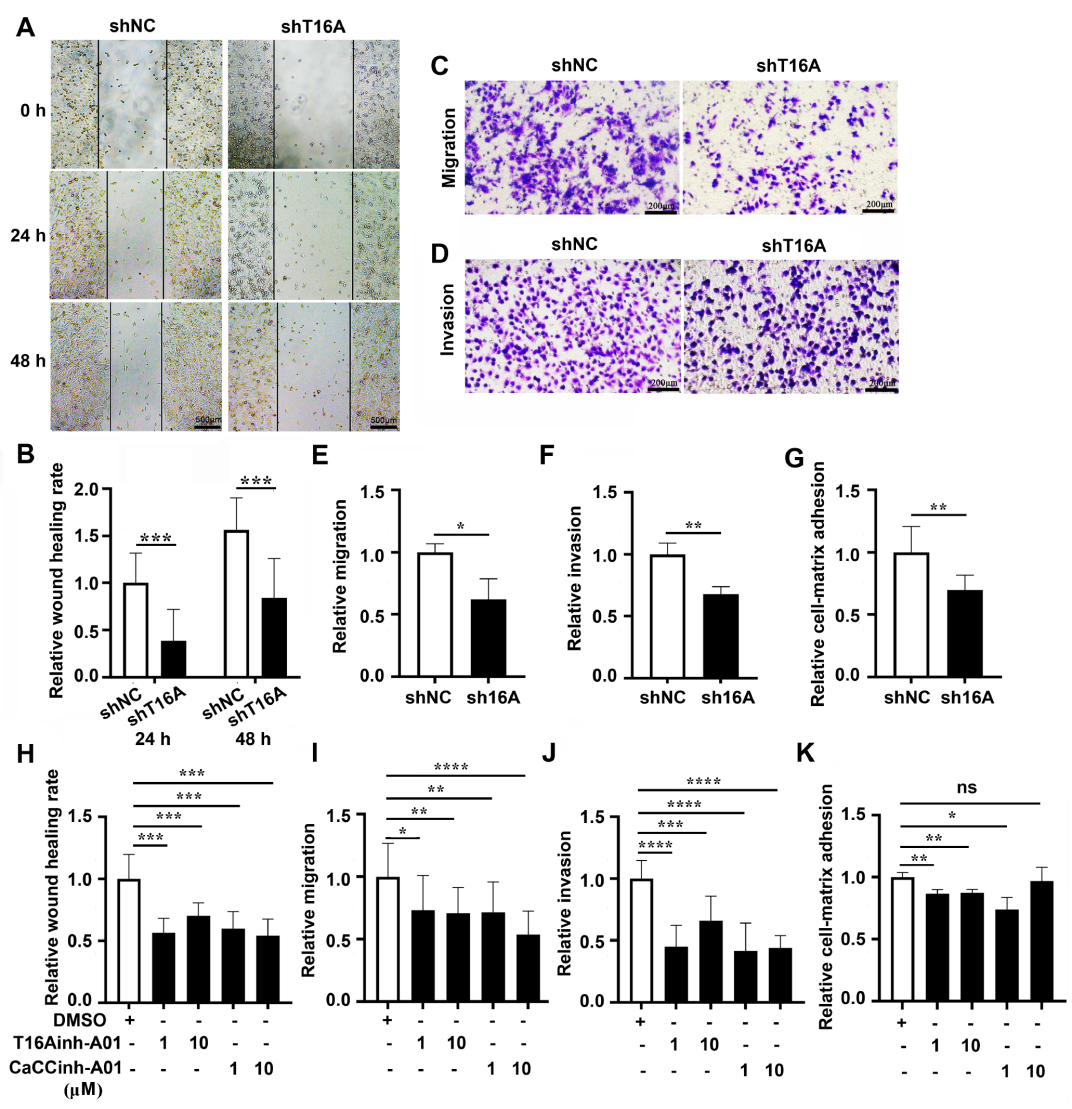
Figure 1 TMEM16A inhibition suppresses melanoma cell migration, invasion, and matrix adhesion (A,B) Wound healing assays in A375 cells transduced with control shRNA (shNC) or shTMEM16A (shT16A) were performed at 24 h and 48 h post-scratching. (C–F) Transwell migration and invasion assays in shNC- and shT16A-transduced A375 cells after 48 h of incubation. (G) Cell-matrix adhesion assays in shNC- and shT16A-transduced A375 cells, as quantified by MTT assays. (H) Wound healing assays of A375 cells treated with T16Ainh-A01 (1 μM, 10 μM) or Caccinh-A01 (1 μM, 10 μM) for 24 h. (I,J) Transwell migration and invasion assays of A375 cells treated with T16Ainh-A01 or Caccinh-A01 (1 μM, 10 μM) for 48 h. (K) Cell-matrix adhesion assay of A375 cells treated with T16Ainh-A01 or Caccinh-A01 (1 μM, 10 μM) for 2 h. Data are presented as the mean ± SD. Statistical significance was determined by Student′s t test. ns, P > 0.05; *P < 0.05; **P < 0.01; ***P < 0.001; ****P < 0.0001. n = 3.

To investigate the molecular mechanisms underlying the effects of TMEM16A, we analyzed the expressions of biomarker genes associated with phenotypic switching and metastasis. The results revealed that the mRNA levels of *N-cadherin*, *Vimentin*, and matrix metalloproteinase 9 ( *MMP9*) were significantly lower in shT16A A375 cells than in shNC control cells ( Supplementary Figure S1). Western blot analysis confirmed consistent decreases at the protein levels. These findings demonstrate that TMEM16A promotes melanoma metastasis by regulating these critical biomarkers.

Given the significant prometastatic effects of TMEM16A *in vitro*, we next evaluated its role in a pulmonary metastasis model. We established a model by intravenous injection of shT16A- or shNC-transfected A375 cells (1 × 10 ^6^ cells/mouse) *via* the tail vein into BALB/c nude mice ( *n*  = 7 per group). All animal experiment procedures were approved by the Institutional Animal Care and Use Committee of Nanjing Normal University (IACUC-20230803). After 30 days, the shNC group presented prominent metastatic nodules on the lung surface, whereas the shT16A group presented no visible nodules ( Figure 2A). Hematoxylin and eosin (H&E)-stained lung sections revealed an 88% reduction in the metastatic foci count (shT16A group: 0.4 ± 0.8 vs shNC group: 5.5 ± 3.4; *P < *0.01) and a significantly decreased metastatic area ( Figure 2B; *P*  < 0.05). These findings conclusively demonstrate that TMEM16A drives melanoma metastasis *in vivo*, whereas its genetic suppression effectively inhibits metastatic progression ( Figure 2C,D).

**Figure 2:**
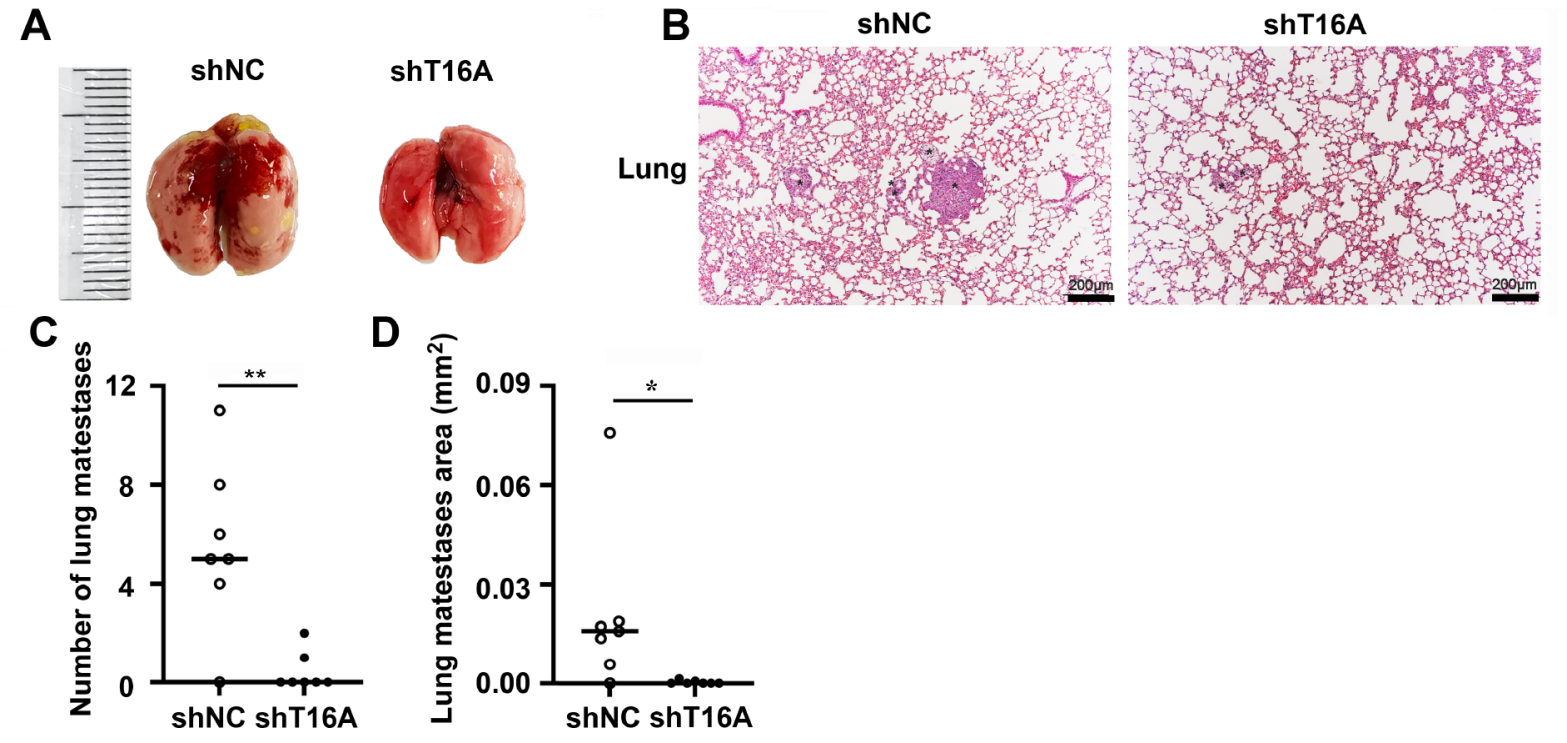
Figure 2 *TMEM16A* knockdown suppresses the lung metastasis of melanoma cells in a mouse model (A) Representative macroscopic lung images from mice injected with shNC- or shT16A-transduced A375 cells. Yellow tumor nodules were visible on the surface of the lungs of the shNC control mice. (B) H&E-stained lung tissue sections from the shNC and shT16A groups. *indicates the metastatic foci. (C,D) Quantification of the number of metastatic foci and total metastatic area. Data are presented as the mean ± SD. Statistical significance was determined by Student’s t test. *P < 0.05; **P < 0.01. n = 7.

To elucidate the molecular mechanisms underlying the role of TMEM16A in promoting melanoma metastasis, we performed comprehensive gene expression profiling of shT16A- and shNC-transduced cells. mRNA expression analysis (volcano plot in Supplementary Figure S2A,B) revealed 38 downregulated and 128 upregulated genes in the shT16A-transduced A375 cells compared with the control cells. Quantitative reverse transcription PCR (qRT-PCR) confirmed the downregulation of multiple oncogenes ( *C1QTNF7*, *CLEC18C*, *MAGED4*, *PIWIL2* and *CD34*) ( Supplementary Figure S2 C‒G). To characterize signaling pathways associated with downregulated differentially expressed genes (DEGs), we performed Gene Ontology (GO) and KEGG pathway enrichment analyses. The results revealed significant alterations in multiple signaling pathways, particularly in cell adhesion, with notably elevated integrin β2 (ITGB2) expression as a featured alteration ( Supplementary Figure S2H‒K). ITGB2 expression was significantly higher in metastatic patients than in primary melanoma patients ( *P*  < 0.001) ( Supplementary Figure S2J). To test whether the elevated expression of ITGB2 is caused by TMEM16A, we measured ITGB2 expression in shT16A-transduced A375 cells. As expected, TMEM16A knockdown significantly reduced both ITGB2 mRNA and protein levels ( Supplementary Figure S2L–N; *P*  < 0.05 and *P*  < 0.01, respectively). Database analyses (UALCAN/ENCORI) revealed positive correlations between TMEM16A and target genes (with C1QTNF7/CD34 showing the strongest associations) ( Supplementary Figures S3 and S4). These results demonstrate that TMEM16A-mediated regulation of ITGB2 contributes to melanoma metastasis.

Given that ITGB2 is known to cooperate with EGFR in activating metastasis-associated pathways ( *e*. *g*., MAPK and AKT) [11], we examined the signaling modules of these pathways. shT16A-transduced A375 cells presented significantly reduced levels of phosphorylated EGFR and phosphorylated AKT. However, the total protein levels of both EFGR and AKT did not significantly change ( Supplementary Figure S5A–E). These data suggest that the *TMEM16A* knockdown-induced attenuation of EGFR/MAPK signaling likely stems from ITGB2 downregulation rather than altered expression of EGFR or AKT themselves.

To evaluate the role of AKT in melanoma metastasis, we treated A375 cells with MK2206, a specific AKT inhibitor, and observed that AKT inhibition significantly suppressed their migration, with a more pronounced effect on shT16A-transduced cells ( Supplementary Figure S5F,G). The enhanced suppression observed in shT16A cells suggests a potential synergistic anti-metastatic effect between AKT inhibition and *TMEM16A* knockdown.

In summary, our study demonstrated that TMEM16A promotes melanoma metastasis through EGFR/AKT-mediated phenotypic switching and the upregulation of migration- and invasion-associated proteins. TMEM16A not only contributes to host immunity [8] but also modulates the tumor microenvironment by regulating metastasis-associated gene expression. On the basis of our previous and current findings, we propose that TMEM16A is a promising therapeutic target for treating both melanoma growth and metastasis.

## Supporting information

25299Supplementary_Data

## Supplementary Data

Supplementary data is available at *Acta Biochimica et Biophysica Sinica* online.
