# Supplementary material for: TMEM16A inhibition suppresses melanoma metastasis: TMEM16A inhibition suppresses melanoma metastasis
Source: Acta Biochim Biophys Sin (Shanghai). 2025 Aug 5;58(3):704–7. doi: 10.3724/abbs.2025133 (PMC13059781; doi:10.3724/abbs.2025133)
Supplement: 25299Supplementary_Data [file 25299Supplementary_Data.docx]

**
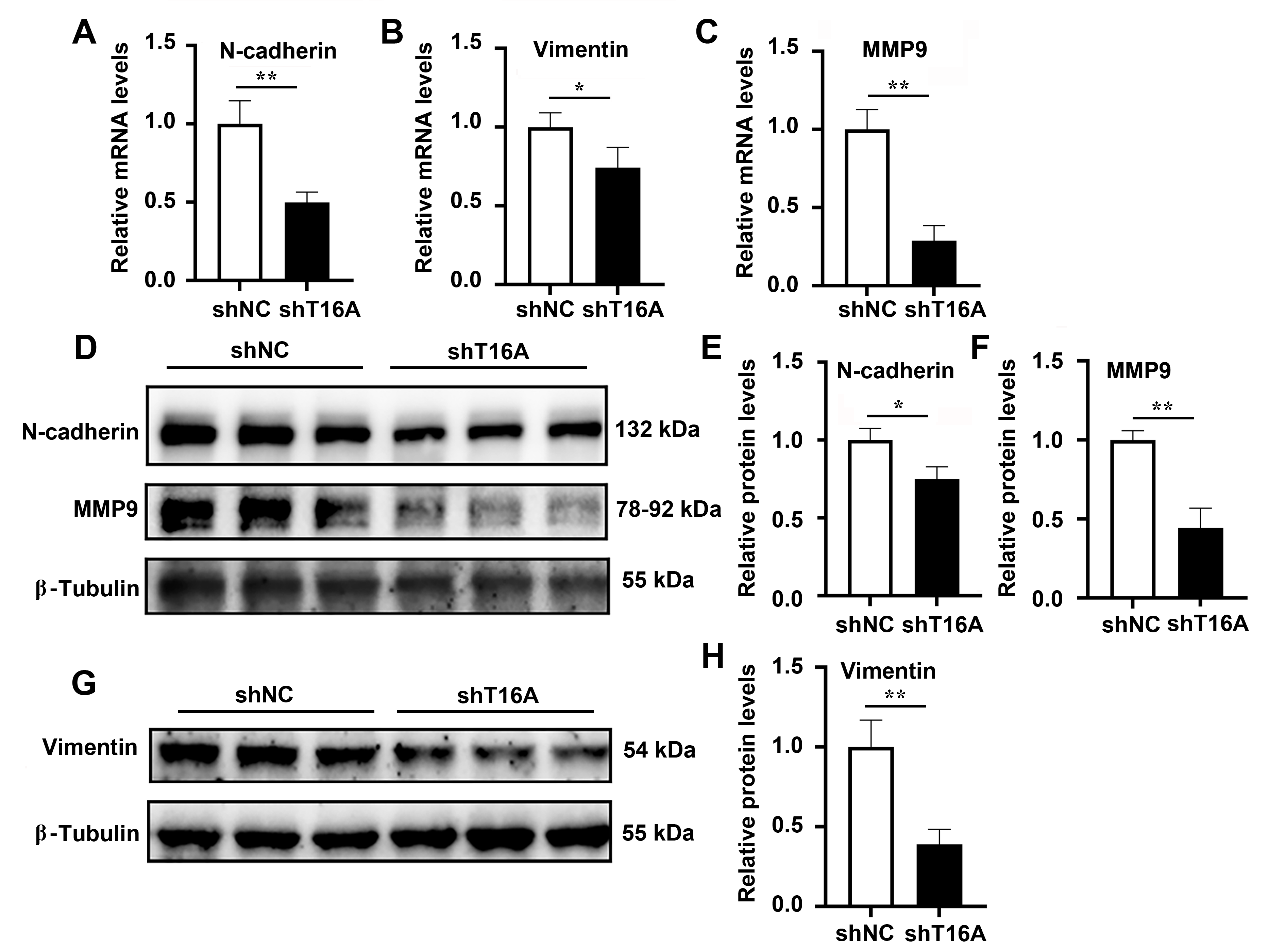
**

**Supplementary Figure S1.** ***TMEM16A* knockdown suppresses metastasis-associated gene expression in A375 melanoma cells**  (A−C) qRT-PCR analysis of mRNA expressions of *N-cadherin*, *Vimentin* and *MMP9* in shNC and shT16A transduced A375 cells. (D−H) Protein expressions detected by western blot analysis. Data are presented as the mean ± SD. Statistical significance was determined by Student’s *t*-test. ns, *P* > 0.05; **P* < 0.05; ***P* < 0. 01. *n* = 3.

**
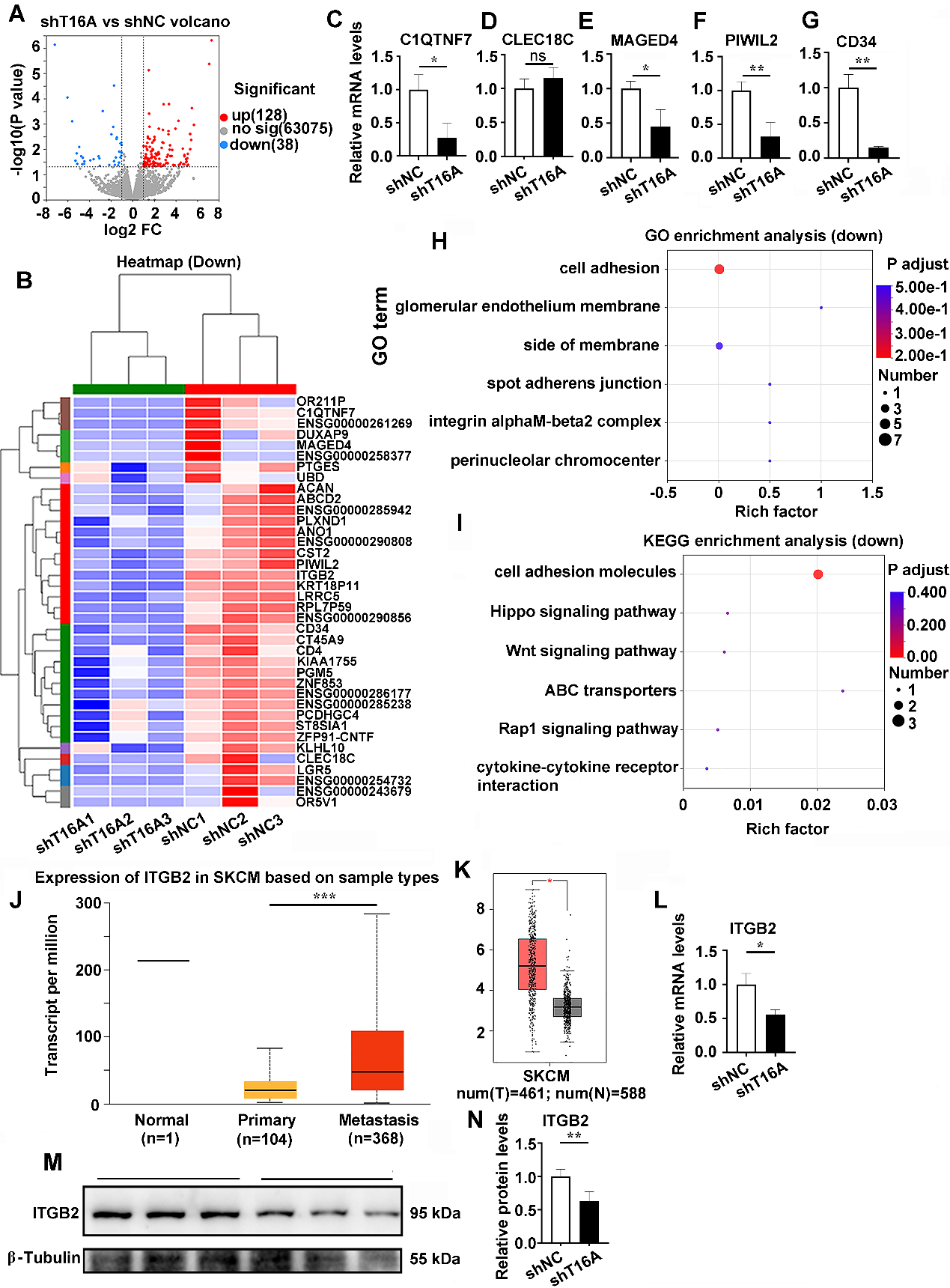
**

**Supplementary Figure S2. *TMEM16A* knockdown alters oncogene expression in melanoma cells** (A) Volcano plot of RNA sequencing data comparing shNC- and shT16A-transduced A375 cells. (B) Heatmap showing downregulated genes in shT16A-transduced A375 cells compared to shNC controls. (C−G) qRT-PCR analysis of *C1QTNF7*, *CLEC18C*, *MAGED4*, *PIWIL2*, and *CD34* expressions in shT16A- and shNC-transduced A375 cells. (H,I) Gene Ontology (GO) and KEGG pathway analyses of downregulated genes (*P* < 0.05, significantly enriched terms/pathways) in shT16A-transduced versus shNC-transduced A375 cells. (J,K) ITGB2 expression in normal tissues versus melanoma (GEPIA: http://gepia.cancer-pku.cn/; UALCAN: http://ualcan.path.uab.edu/index.html). (L−N) ITGB2 mRNA and protein expression in shNC and shT16A transduced A375 cells. Data are presented as the mean ± SD. Statistical significance was determined by Student’s *t-*test: ns, *P* > 0.05; * *P* < 0.05; ***P* < 0.01; ****P* < 0.001. *n* = 3.


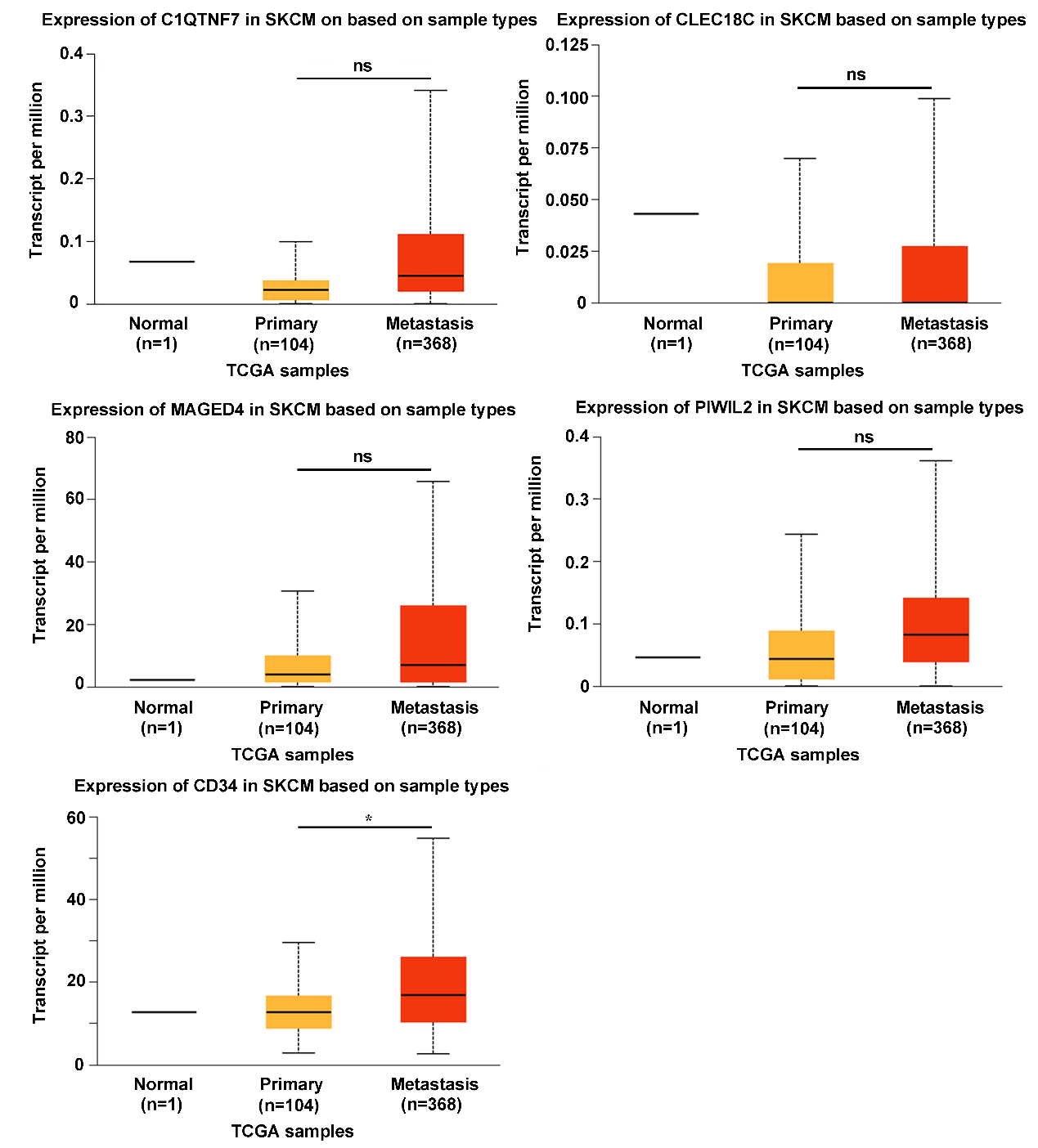


**Supplementary Figure S3. Expressions of *C1QTNF7*, *CLEC18C*, *MAGED4*, *PIWIL2*, and *CD34* in normal tissues, primary melanoma and metastatic melanoma** Data are obtained from UALCAN: http://ualcan.path.uab.edu/index.html). ns, *P* > 0.05; **P* < 0.05.


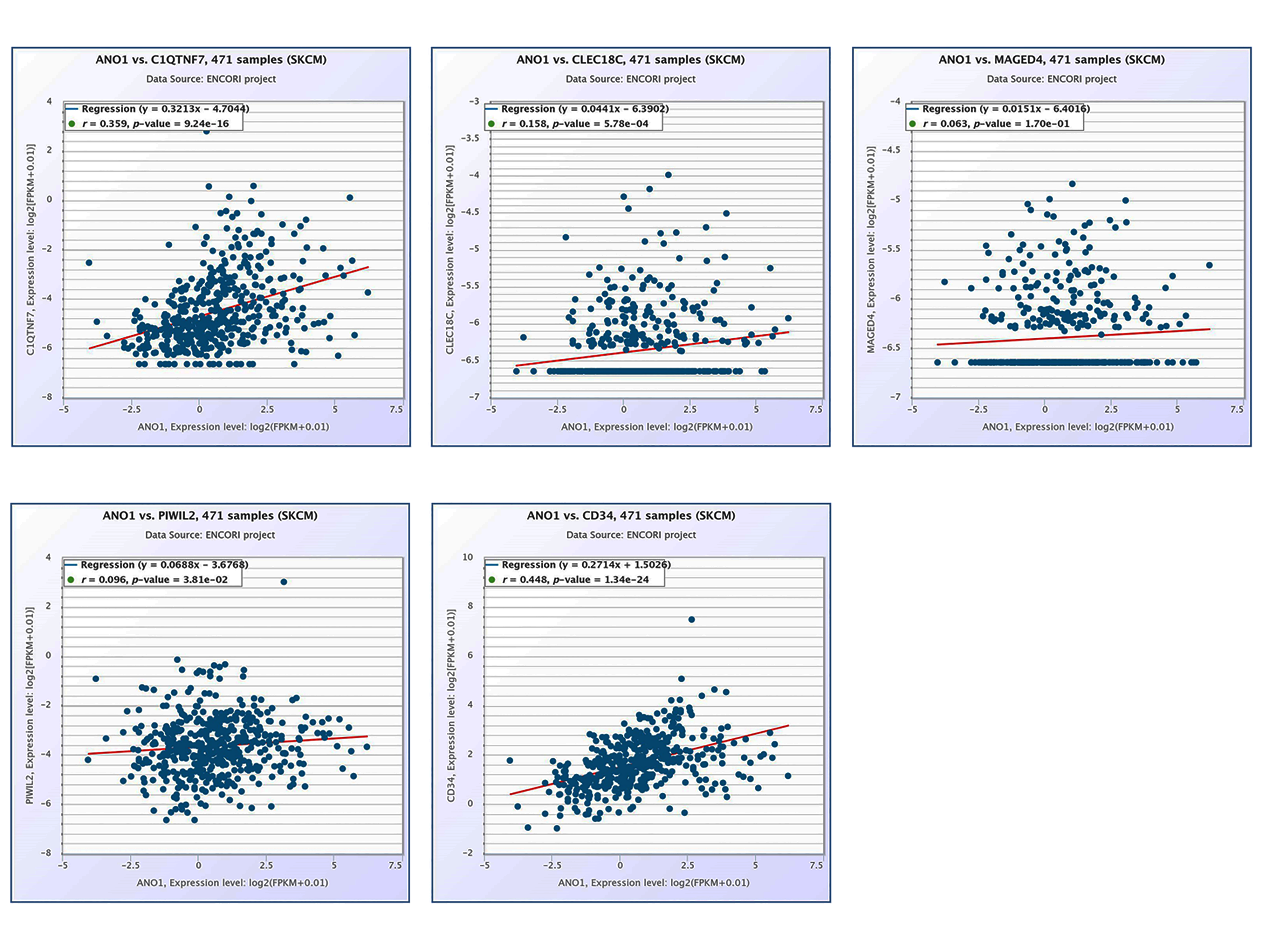


**Supplementary Figure S4. Correlation between C1QTNF7, CLEC18C, MAGED4, PIWIL2, CD34 and TMEM16A (ANO1) expressions** Data are obtained from the ENCORI/starBase: https://rnasysu.com/encori/.

**
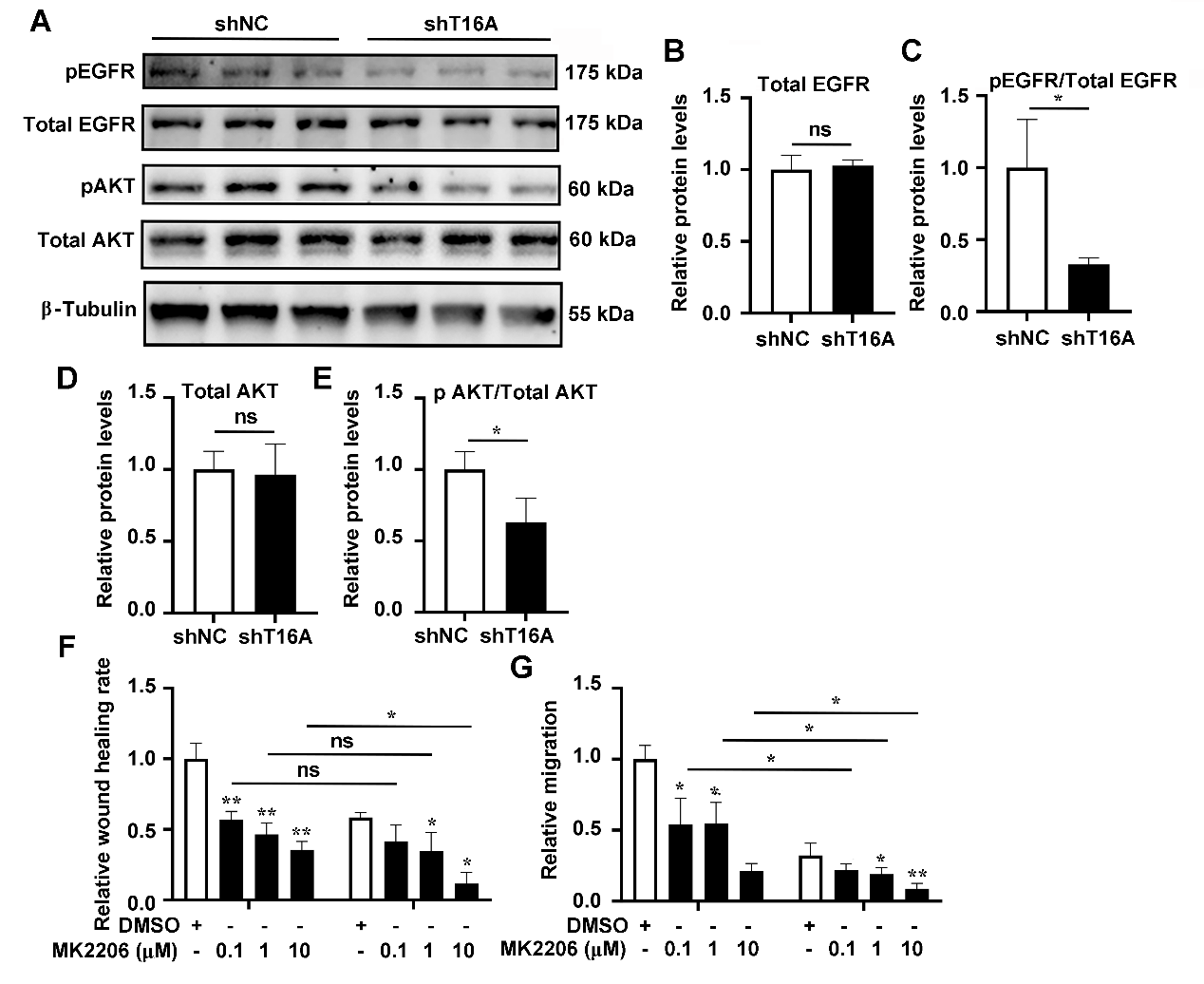
**

**Supplementary Figure S5. *TMEM16A* knockdown attenuates the EGFR/AKT signaling pathway** (A−E) Western blot analysis of pEGFR and pAKT levels in shNC- and shT16A- transduced A375 cells. (F) Wound healing assays of shNC- and shT16A-transduced A375 cells treated with or without MK-2206 (0.1, 1, or 10 μM) at 24 h post-scratching. (G) Transwell migration assays of shNC- and shT16A-transduced A375 cells treated with or without MK-2206 (0.1, 1, or 10 μM) at 48 h post-incubation. Data are presented as the mean ± SD. Statistical significance was determined by Student’s *t*-test. ns, *P* > 0.05; **P* < 0.05; ***P* < 0.01; ****P* < 0.001. *n* = 3.

**Supplementary Table S1. Sequences of primer for qRT-PCR in this study**

| Gene | Forward primer (5′→3′) | Reverse primer (5′→3′) |
| --- | --- | --- |
| *GAPDH* (H) | CAGGAGGCATTGCTGATGAT | GAAGGCTGGGGCTCATTT |
| *TMEM16A* (H) | GAGCCAAAGACATCGGAATCTG | TGAAGGAGATCACGAAGGCAT |
| *E-cadherin* (H) | AAGGCACGCCTGTCGAAGCA | ACGTTGTCCCGGGTGTCATCCT |
| *N-cadherin* (H) | TGCGCGTGAAGGTTTGCCAGT | TGGCGTTCTTTATCCCGGCGT |
| *Vimentin* (H) | ACCGCACACAGCAAGGCGAT | CGATTGAGGGCTCCTAGCGGTT |
| *MMP-9* (H) | TGGGCTACGTGACCTATGAC | CAAAGGTGAGAAGAGAGGGC |
| *C1QTNF7* (H) | TGAAAAGGGAACTGCAGGTTTG | ATGCCAACAGAAAAGGCGGA |
| *CLEC18C* (H) | GTGCGTCTGTGACATCGGCTAC | CCTCTGACATTTCATCCTGGCTCTG |
| *MAGED4* (H) | TCGCCTCCTTGGAAAAACCA | AGTCCCATCTTGCGTAGTGC |
| *PIWIL2* (H) | GGCCCA AGTGGACGTCTTA | TTCCCCCTGGGATCAGGTAG |
| *CD34* (H) | CAACACCTAGTACCCTTGGAAGT | ACTGTCGTTTCTGTGATGTTTGT |
| *ITGB2* (H) | TGCGTCCTCTCTCAGGAGTG | GGTCCATGATGTCGTCAGCC |
| *GAPDH* (M) | ACTCCCACTCTTCCACCTTC | TCTTGCTCAGTGTCCTTGC |
| *E-cadherin* (M) | GGTTTTCTACAGCATCACCG | GCTTCCCCATTTGATGACAC |
| *N-cadherin* (M) | TGAAACGGCGGGATAAAGAG | GGCTCCACAGTATCTGGTTG |
| *Vimentin* (M) | CGGCTGCGAGAGAAATTGC | CCATTTCCGTTCAAGGTCAAG |
| *MMP-9* (M) | AGGTGCCTCGGATGGTTATCG | TGCTTGCCCAGGAAGACGAA |

H: human, M: mouse.
